# Procyanidin-Rich Extract from Grape Seeds as a Putative Tool against *Helicobacter pylori*

**DOI:** 10.3390/foods9101370

**Published:** 2020-09-26

**Authors:** Jose Manuel Silvan, Alba Gutiérrez-Docio, Silvia Moreno-Fernandez, Teresa Alarcón-Cavero, Marin Prodanov, Adolfo J. Martinez-Rodriguez

**Affiliations:** 1 Microbiology and Food Biocatalysis Group, Department of Biotechnology and Food Microbiology, Institute of Food Science Research (CIAL, CSIC-UAM), C/Nicolás Cabrera, 9. Cantoblanco Campus, Autonoma University of Madrid, 28049 Madrid, Spain; 2 Department of Production and Characterization of Novel Foods, Institute of Food Science Research (CIAL, CSIC-UAM), C/Nicolas Cabrera 9. Cantoblanco Campus, Autonoma University of Madrid, 28049 Madrid, Spain; alba.gutierrez@uam.es (A.G.-D.); silvia.moreno@uam.es (S.M.-F.); marin.prodanov@uam.es (M.P.); 3 Microbiology Department, Hospital Universitario de La Princesa, Sanitaria Princesa Research Institute, 28006 Madrid, Spain; talarcon@helicobacterspain.com; 4 Department of Preventive Medicine, Public Health and Microbiology, School of Medicine, Autonomous University of Madrid, 28049 Madrid, Spain

**Keywords:** grape seed extract, procyanidins, *Helicobacter pylori*, antibacterial activity, antibiotic resistance

## Abstract

Strains of *Helicobacter pylori (H. pylori)* resistant to various antibiotics have increased in recent years. In this context, the search for new therapeutic approaches is crucial. The aim of the present study was to demonstrate the antibacterial activity of a procyanidin-rich extract obtained from food-grade winery grape seeds against 14 *H. pylori* strains and elucidate its phenolic composition. Ten strains (71.4%) showed resistance to at least some of the tested antibiotics, while four isolates (28.6%) were susceptible to all antibiotics. Resistance to more than one class of antibiotics was observed in six strains (42.9%). The extract was able to inhibit the growth of all *H. pylori* strains in a range of a minimum inhibitory concentration (MIC) from 0.015 mg/mL to 0.125 mg/mL, confirming also the existence of a strain-dependent effect. The phenolic composition determined by reverse phase high pressure liquid chromatography, photodiode array, and mass spectrometry detection (RP-HPLC-PAD-MS) analysis revealed the presence of 43 individual compounds and allowed the quantification of 41 of them, including seven procyanidin tetramers, seven procyanidin pentamers, and six galloylated procyanidin dimers, trimers, and tetramers. The extract was composed mainly by catechin and procyanidin oligomers with a total amount of 5801 mg/100 g, which represent 92% of the total individual phenolic content. Among them, the most abundant were catechins (2047 mg/100 g), followed by procyanidin dimers (1550 mg/100 g), trimers (1176 mg/100 g), tetramers (436 mg/100 g), and pentamers (296 mg/100 g) that represent 35, 27, 20, 8, and 5%, respectively of the total flavanol constituents. The composition profile information may help to improve the production process of useful antibacterial extracts against *H. pylori*.

## 1. Introduction

*H. pylori* is a Gram-negative spiral rod bacterium that colonizes the gastric mucosa, producing an inflammatory response. It is known as the most common human pathogen infecting more than 50% of the world’s population and causes a severe problem when the infection aggressively promotes gastric cancer progression [1]. Early eradication-based therapies have been proven to regress *H. pylori*-associated damages. When treatment is needed, a first-line standard triple therapy is currently employed, which use to comprise two of three antibiotics, including amoxicillin, clarithromycin, and metronidazole combined with one proton pump inhibitor (PPI). Nevertheless, the use of levofloxacin in triple therapy and bismuth-based quadruple therapy have also been suggested as second-line therapies following the failure of the clarithromycin-containing treatments [2]. Furthermore, tetracycline and rifampicin are among the common antibiotics that have been used in several rescue therapies recommended in the eradication of *H. pylori* infection [3,4]. However, the efficacy of eradication treatments has been extremely compromised primarily because of the increased resistance to antibiotic agents [5]. Therefore, due to the high increase of *H. pylori* resistance to the antibiotics used for its treatment, the search for natural and sustainable alternatives to the use of antibiotics is a serious challenge. It would be a promising tool to incorporate new therapeutic practices against this pathogen, reducing the high antibiotic dose of the current treatments and providing an alternative for 20% of infected people with symptoms (140 million people worldwide) [6] for which antibiotic treatment is ineffective, thus contributing to improve population health. In this regard, there is a growing interest in the use of natural antibacterial compounds, such as plant extracts, rich in phenolic compounds.

Grape seeds are obtained from grape pomace, a by-product from the wine industry. With an average production of 5,500,000 tons per year, Spain stays among the first three most important grape producers in the world. The biggest part of this production gives an average of 3,921,900,000 L of wine and grape must per year (mean value of the last five vintages) and 210,000,000 L of lees and 560,000 tons per year of pomace, a solid by-product, composed by seeds, skins, and stems [7]. The most valuable components of this by-product are sugars (40–55 g/kg) and tartaric acid salts (1–1.5 g/kg) that are the main row material for the distilleries of wine alcohol. That is why, at the end of the winemaking campaign, the grape pomace produced in the wineries and the grape juice producers ends up and accumulates into the distilleries for alcohol and tartaric salt recovery. This activity produces the same volume of spent pomace and 1 to 1.3 times bigger volumes of waste waters (vinasses) [8,9], which are the main ecological problems of these industries [10]. The spent pomace is dried, and the seed fraction is separated and sent to the oil producers for oil recovery. Due to the direct contact of the pomace with the drying agent during dehydration (in most cases, hot gases from biomass combustion boilers with temperatures around 850 °C), the seeds are highly contaminated with Maillard reaction degradation products that makes refining of the oil mandatory for its use as a food. The rests of the pomace (skins and stems), plus the defatted seed paste are used usually as biomass for industrial production of heat (energy) or still lower added value soil amendment products [8,11].

From industrial point of view, grape seeds are one of the most important natural renewable resources of catechins and procyanidins, because of their relative abundance and low cost. The main interest of these compounds resides in their high reducing (antioxidant) activity [12], that has been found to be 20- and 50-times higher than those of the most studied natural antioxidants, vitamins C and E, respectively [13]. Among others bioactive properties attributed to catechins and procyanidins, they have been associated to antibacterial properties against several pathogenic bacteria [14,15]. They can inhibit the growth of a broad spectrum of Gram-negative [16,17,18] and Gram-positive [19,20,21] bacteria, depending on its concentration, type of phenolic compounds presents in the extract, bacterial species, and tested strains.

**Figure 1:**
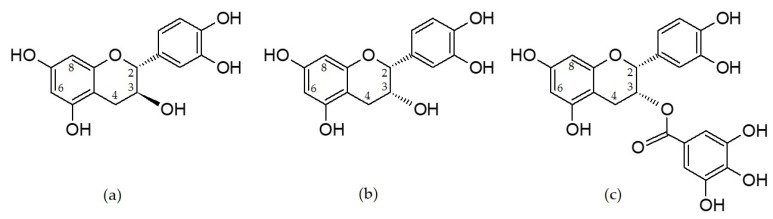
Molecular structures of (+)-catechin (**a**), (−)-epicatechin (**b**) and (−)-epicatechin gallate (**c**).

Grape seed procyanidins, known also as condensed tannins, are a sub-class of polyphenols with extremely divers structures in spite of that they are based only on three elemental flavan-3-ol units: (+)-catechin, (−)-epicatechin, and (−)-epicatechin gallate (Figure 1).

This diversity is due to some of their particular features: the stereochemistry of the asymmetric carbon atoms C2 and C3 of the flavan skeleton, type of interflavan bond (C4-C8 or C4-C6), the length of the polymer chain (degree of polymerization), the degree of galloylation and the position of the gallic acid ester in the polymer chain [22] and their ability to form complex structures with other biopolymers, polysaccharides and proteins [23]. An excellent description of these structures is reviewed by Dixon et al. [24]. However, this enormous diversity makes their assessment extremely difficult. In fact, there is not an analytical method able to solve this problem completely. In general, procyanidins are analyzed by reverse (RP) and/or normal phase high pressure liquid chromatography (HPLC) or ultra-high pressure chromatography (UPLC), or two-dimensional HPLC, coupled to photodiode array (PAD) and different kinds of mass spectrometry (MS) detectors, but only some procyanidins have been separated (up to heptamers) and quantified (up to tetramers) in this way [25,26,27]. It is only recently when the development of some multi-model regression tools for computational analysis of wide range of analytical parameters acquired by UPLC coupled to adrift tube ion mobility MS detector were capable to discriminate among co-eluting procyanidin ions and allowed Li et al. [28] to do an important leapfrogging by the simultaneous characterization of up to 686 procyanidins with degree of polymerization of up to 15. Nevertheless, it should be highlighted that these results should be taken with precaution, as their identification is only tentative.

For procyanidin recovery, the choice of the most proper extraction conditions is critical for the quality of the final product. A large number of studies dedicated on extraction of catechins and procyanidins from different vegetable sources [29,30,31] put in evidence that methanol, acetone and their mixtures with water at proportions from 0 to 30% of water are most used solvents. Nevertheless, when the extracts are destined to food additives, the use of these solvents is not appropriate, due to the possible toxic effect of the residues that can remain in the final product. In these cases, the alternatives are water, ethanol and/or hydroalcoholic mixtures. Due to the high polarity nature of procyanidins, they can be extracted only by water. The main problem in this case is the low extraction yields of procyanidins, because an important part of them remains tightly bound to the plant cellular wall [32]. Improving their extractability requires an intensification of the process, which is usually done by increasing the internal energy of the system with ultrasound agitation or heating. Power ultrasound energy is quite effective for accelerating analytical and preparative extractions [33,34], but is quite limited for big industrial applications, because requires constant recirculation of the extraction slurry around the ultrasound emitting probs at very low flows. Another problem is the co-extraction of other seed constituents, such as saccharides and proteins, at these conditions of intensification, and consequently, the need for further purification of the extract, besides formation of unwanted compounds that results from heating. Addition of less polar solvents, such as ethanol, to the water, improves considerably extraction yields of procyanidins and suppresses saccharide and protein hydration and their further diffusion to the extract to an important grade (not published data). Therefore, real alternatives for industrial procyanidin extraction are only water and/or hydroalcoholic mixtures and heating. In this sense, it is important to note that use of heat must be moderate, avoiding temperatures higher than 50 °C, because of the formation of unwanted degradation products.

Therefore, it becomes obvious that leading way to increase the global grape by-product usefulness goes through the improvement of the transformation technology and creation of innovative products with higher added values [35]. For these reasons, the aim of this study was to demonstrate the antibacterial efficiency of a procyanidin-rich extract from food-grade winery grape seeds against 14 *H. pylori* strains and elucidate its phenolic composition.

## 2. Materials and Methods

### 2.1. Materials

Food-grade pomace from white *Vitis vinifera* L. grapes (variety Airén) was taken immediately after their discharge from an industrial pneumatic press located in the winery Virgen de las Viñas (Argamasilla de Alba, Spain) and was placed in a discontinuous dryer with a forced hot air circulation (Drybig, Selecta, Spain). The pomace was spread out in thin layers on stainless steel mesh trays and dried at 50 °C for 15 h. Grape seeds were separated by conducting the dry pomace into vibrating screen separator (Industrias Joan Busquets Crusat S.A., Reus, Spain). This separator was provided with a cyclone system that allows eliminating light particles, such as fine pieces of skins, very small seeds, peduncles, including fine dust from the main seed fraction. Finally, a purity of 99.5% of grape seeds was obtained.

For grape seed extraction, demineralized water with electrical conductivity of 5 μS/cm was obtained in-house by a reverse osmosis unit (Genius 300, Filtec Depuradoras, Girona, Spain). For HPLC analysis, milli-Q grade water was obtained in-house by a Milli-Q^®^ Integral 3 purification system (Merck Millipore, MA, USA). HPLC grade methanol and acetonitrile were purchased from Scharlab (Barcelona, Spain) and glacial acetic acid, from Sigma-Aldrich (Madrid, Spain). For HPLC peak identification, the following reference substances were used: HPLC grade (+)-catechin (C), (−)-epicatechin (EC), (−)-epicatechin-3-gallate (ECG), procyanidin dimers B_1_ [EC-(4α-8)-C], B_2_ [EC-(4α-8)-EC] and B_3_ [C-(4α-8)-C], 3,4-dihydroxy benzoic acid (3,4-DHBA or protocatechuic acid), *trans*-caftaric acid, quercetin-3-O-glucuronide and quercetin-3-O-glucoside were purchased from Extrasynthèse (Genay, France). HPLC grade 3,4,5-trihydroxybenzoic acid (3,4,5-THBA or gallic acid), methylgallate, ethylgallate, tryptophan and ellagic acid were obtained from Sigma-Aldrich. Procyanidin dimers B_1_–B_8_ were purified previously by high-speed countercurrent chromatography and characterized by nuclear magnetic resonance spectroscopy as previously described [36] and used for dimeric procyanidin identification [37]. Purified procyanidin extract from cocoa (Breko GmbH, Bremen, Germany) was used as a complex reference sample for the identification of procyanidin trimer C_1_ [EC-(4α-8)-EC-(4α-8)-EC], tetramer [EC-(4α-8)-EC-(4α-8)-EC-(4α-8)-EC] and pentamer [EC-(4α-8)-EC-(4α-8)-EC-(4α-8)-EC-(4α-8)-EC] [30].

### 2.2. Elaboration of Procyanidin-Rich Extract from Food-Grade Winery Grape Seeds

Grape seed extract (GSE) was obtained in a pilot-scale solid/liquid extraction unit, provided with a 30 L extraction vessel. A nylon mesh strainer bag with a mean hole size of 300 µm was used to hold the seeds in the extraction vessel. An amount of 4 kg of dried grape seeds was hydrated with 6.5 L of demineralized water for overnight in the extraction unit at room temperature. After hydration, 21.5 L of 96% ethanol were added to the seeds to reach a final concentration of 70% ethanol. The thermostatic system extraction unit was set at 40 ± 2 °C. Extraction was completed during 5 days of maceration of the seeds. Intensification of the extraction was carried out by mechanical stirring of the seeds inside the strainer bag twice per day. An amount of 22.2 L was obtained by free draining (no pressing) of the extract. Two aliquots of 1 L of the crude extract were clarified by centrifugation at 8570× *g* during 20 min and filtered through a glass microfiber filter MFV6 from Letslab (Barcelona, Spain) to obtain 1.1 nephelometric turbidity units (NTU). Turbidity was determined by a model D-112 turbidimeter in the interval of 0 to 800 NTU (Dinko Instruments, Barcelona, Spain). The two clarified aliquots were submitted to distillation in rotavapor R-151 at 60 mbar of pressure for ethanol recovery (Büchi, Labortechnik AG, Flawil, Switzerland). The two ethanol-free water phases were freeze-dried and amounts of 15.22 and 14.98 g of dry mass (dm) were obtained, respectively. The remaining 20 L of extract were dealcoholized by distillation of the ethanol at 60 mbar of pressure by a rotavapor, concentrated afterwards to 26 g/100 mL of total soluble substances at 22 mbar of pressure and kept at 4 °C for a week. At these conditions, a solid sediment was formed and removed by centrifugation at 8570× *g* for 20 min.

Measurement of total soluble substances (dry mass) was carried out by two methodologies. For samples containing ethanol (i.e., the crude GSE), total soluble substances were measured gravimetrically, after freeze-drying of known volume of extract. For aqueous samples (i.e., dealcoholized GSE) total soluble substances were measured by hand-held refractometer Atago (Fukaya, Japan), calibrated in the interval of 0 to 32 g/100 mL (°Brix).

### 2.3. Chemical Characterization of the Procyanidin-Rich Grape Seed Extract

Solutions of 20 and 40 mg/mL of the freeze-dried GSE were prepared with ethanol/water (1/1, *v/v*) (in quadruplicate) and were analyzed quantitatively by reversed phase high pressure liquid chromatography coupled to photo-diode array detector and mass spectrometry detector with electrospray ionization source (RP-HPLC-PAD-MS(ESI)) as previously described [38]. Methylgallate, ethylgallate, tryptophan, 3,4,5-THBA, 3,4-DHBA, *trans*-caftaric acid, quercetin-3-*O*-glucuronide, quercetin-3-*O*-glucoside, ellagic acid, C, EC, ECG, procyanidin dimers B_1_, B_2_, B_3_, B_4_, B_5_, trimer C_1_, tetramer [EC-(4α-8)-EC-(4α-8)-EC-(4α-8)-EC] and pentamer [EC-(4α-8)-EC-(4α-8)-EC-(4α-8)-EC-(4α-8)-EC] were identified unambiguously by co-elution and comparison with the retention time, order of elution, ultraviolet (UV) spectra, and pseudo-molecular and fragment ion masses of the corresponding purified reference substances and purified procyanidin extract from cocoa. The rest of procyanidins were identified tentatively according to their retention time, order of elution, UV spectra, pseudo-molecular and fragment ion masses, and bibliographic data [37,39,40,41]. All above mentioned compounds were quantified using external reference calibration curves, plotted with the corresponding purified reference substances. All non-galloylated procyanidin dimers, trimers, tetramers, and pentamers were quantified in equivalents of procyanidin dimer B_1_ and all galloylated procyanidins from dimers to tetramers were quantified in equivalents of ECG. For peaks that contained two components with similar structures (e.g., PC_2_ and PC_5_) the whole area (amount) of each of them was divided by two and referred to each of them at equal amounts. For peaks that contained two components with different structures (e.g., PC_2_ (B_1_) and methylgallate) the whole area (amount) of the peak was assigned to the compound with the most intense UV spectra and the other was considered as impurity, i.e., its amount was not taken in consideration. Results were presented as mean value (*n* = 4) ± standard deviation (SD) and expressed as milligrams per 100 g of dry mass (mg/100 g dm).

Total phenolic content was determined by the Folin–Ciocalteu assay [16]. The results were expressed as mg of gallic acid equivalents/100 g dm extract. Determination of total procyanidins was carried out by the acid butanol assay [42]. The results were expressed as mg of cyanidin equivalents/100 g dm extract.

### 2.4. Helicobacter pylori Strains, Growth Media, and Culture Conditions

*Helicobacter pylori* strains were isolated from gastric mucosal biopsy obtained from symptomatic patients from the Microbiology Department of Hospital La Princesa (Madrid, Spain). Biopsies were cultured in selective (*Pylori* agar, BioMerieux, Madrid, Spain) and non-selective media (Blood-supplemented Columbia Agar, BioMerieux) obtained commercially. Strains were identified by colony and Gram stain morphology, and urease, oxidase and catalase positive test. *H. pylori* strains were stored at −80 °C in Brucella Broth (BB) (Becton, Dickinson and Company, Madrid, Spain) plus 20% glycerol. The agar-plating medium consisted of Müeller-Hinton agar supplemented with 5% defibrinated sheep blood (MHB) (Becton, Dickinson and Company, Madrid, Spain), and liquid growth medium consisted of BB supplemented with 10% horse serum (HS) (Biowest, Barcelona, Spain). *H. pylori* strains inoculum was prepared as follows: frozen stored strains were reactivated by inoculation (200 μL) in MHB plate and incubation in a microaerophilic atmosphere using a Variable Atmosphere Incubator (VAIN) (85% N_2_, 10% CO_2_, 5% O_2_) (MACS-VA500, Don Whitley Scientific, Bingley, UK) at 37 °C for 72 h. Bacterial biomass grown in one MHB plate was resuspended in 2 mL of BB in a concentration around 1 × 10^8^ colony forming units (CFU)/mL (OD 1.2 at 600 nm and checked retrospectively by viable count) and used as experimental bacterial inoculum in the different experimental assays.

### 2.5. Antibiotic Susceptibility Test

Antibiotic susceptibility of isolated *H. pylori* strains was performed by the E-test (BioMérieux) determining the minimum inhibitory concentrations (MICs) against amoxicillin, clarithromycin, levofloxacin, metronidazole, rifampicin, and tetracycline. A bacterial suspension was prepared in BB supplemented with 10% HS and 200 μL of this suspension was transferred onto the surface of the MHB and streaked with a cotton swab. Antibiotic strips were applied onto the surface of inoculated and dried agar plates. The plates were incubated in a microaerophilic incubator (VAIN) at 37 °C for 72 h before examination. MIC was determined by considering the point where ellipse growth cut with the scale number in the E-test strip. The breakpoints were defined as follows: amoxicillin, MIC > 0.125 μg/mL; clarithromycin, MIC > 0.5 μg/mL; levofloxacin, MIC > 1 μg/mL; metronidazole, MIC > 8 μg/mL; rifampicin, MIC > 1 μg/mL; and tetracycline, MIC > 1 μg/mL, following the European Committee on Antimicrobial Susceptibility Testing (EUCAST) guidelines (version 8.0). Strain Hp11637 (NCTC) was used as experimental control.

### 2.6. Antibacterial Activity

The antibacterial activity of the procyanidin-rich extract against *H. pylori* strains was evaluated following the procedure described by Silvan et al. [16]. Briefly, 1 mL of the extract (2 mg/mL final concentration) was transferred in flasks containing 4 mL of BB supplemented with 10% HS. Bacterial inoculum (50 μL of ~1 × 10^8^ CFU/mL) was then inoculated into the flasks under aseptic conditions. The cultures were prepared in triplicate and incubated under stirring (150 rpm) in a microaerophilic atmosphere using a VAIN at 37 °C for 48 h. Growth controls were prepared by transferring 1 mL of sterile water to 4 mL of BB supplemented with 10% HS and 50 μL of bacterial inoculum. After incubation, serial decimal dilutions of the mixtures were prepared in saline solution (0.9% NaCl) and they were plated (20 μL) onto fresh MHB agar and incubated in a microaerophilic atmosphere using a VAIN at 37 °C for 72 h. Strain Hp11637 (NCTC) was used as experimental control. The number of CFU was assessed after incubation and results were expressed as log_10_ CFU/mL. MIC was determined following the procedure described above and by using GSE diluted in BB to obtain the desired final concentrations. MIC was defined as the lowest amount of extract that provokes a significant decrease (*p* < 0.05) in viability respect to the control growth after 48 h of treatment. The dilution intervals for determination of MIC ranged from 0.015 mg/mL to 2 mg/mL. % growth reduction was calculated by Equation (1):

% reduction = (A − B)/A × 100(1)

A = average value of CFUs of untreated sample

B = average value of CFUs of treated sample

### 2.7. Statistical Analysis

The results for bioactivity assays were reported as mean values ± SD of at least three determinations. A *t*-test was used to assess the differences in antibacterial activity. Differences were considered significant at *p* < 0.05. All statistical tests were performed with IBM SPSS Statistics for Windows, Version 25.0 (IBM Corp., Armonk, New York, NY, USA).

## 3. Results

### 3.1. Physicochemical Characterization of Procyanidin-Rich Grape Seed Extract

An amount of 22.2 L of crude GSE with moderate turbidity (386 NTU) was obtained. An attempt for direct cold clarification of the extract (overnight, at 4 °C) had no visible effect on suspended solid sedimentation. The extract was concentrated to 26 g/100 mL of total soluble substances and submitted again to clarification at 4 °C for a week. In these conditions, insoluble solids settled quite good and allowed obtaining a particle-free (clarified) GSE after centrifugation with turbidity of 0.8 NTU. With respect to the total soluble substances of the crude extract, a mean value of 15.12 g/L was obtained that corresponds to quite elevated (8.39%) extraction yield (with respect to the grape seed material). Another peculiarity of the production of this extract was the use of whole grape seeds, which is an unusual procedure in the industrial production of GSE [43]. This may lead to some incomplete extraction of procyanidins, but it does improve all post extraction treatments of the crude extract, such as clarification and oil separation, and has decisive contribution in the production of completely soluble and translucent GSE when dissolved in water.

Regarding the phenolic composition of the extract, RP-HPLC-PAD-MS analysis revealed the presence of 43 individual compounds, including seven procyanidin tetramers, seven procyanidin pentamers, and six galloylated procyanidin dimers, trimers, and tetramers (Table 1).

The individual separation and identification of a high number of procyanidin oligomers with higher degree of polymerization (tetramers and pentamers) within one analytical run and a standard HPLC equipment could be attributed mainly to the increased analytical resolution (3 µm particle size and 20 cm length) and improved selectivity of the used stationary phase (C18 AR—highly specific for compounds with aromatic functionality), the longer analytical time (120 min) and the singularity of the grape variety Airén.

From qualitative point of view, the results shown in Table 1 are consistent with others described previously [37,38,40,44,45], with the exception of ethylgallate, which was identified at concentration of 179 mg/100 g for first time. Up to now, only the gallic acid derivative methylgalate was identified as naturally present in GSE [37]. Therefore, it seems more likely that ethylgallate should be a product of reaction between gallic acid (which is always present in GSE) and ethanol, created during the maceration of the seeds. If it is so, it becomes obvious that even such mild conditions of extraction were used in the present study, they could be associated to modifications in grape components. This highlights the importance of parameters such as extraction temperature or time in the final composition of the extract.

From quantitative point of view, the results shown in Table 1 indicate that the extract was composed mainly by catechins and procyanidin oligomers (OPC) with a total amount of 5801 mg/100 g dm, which represent 92% of the total individual phenolic content of the extract. Among them, the most abundant were catechin monomers (2047 mg/100 g), followed by procyanidin dimers (1550 mg/100 g), trimers (1176 mg/100 g), tetramers (436 mg/100 g) and pentamers (296 mg/100 g dm) that represent 35, 27, 20, 8, and 5%, respectively of the total flavanol constituents. In all these groups, the amounts of the non-galloylated procyanidins were higher than those of the corresponding galloylated forms. Regarding individual catechins and procyanidins, major compounds were catechin, procyanidin dimers B_1_, B_2_ and B_3_, epicatechin, and two procyanidin trimers (one co-eluting with EC and C_1_) with amounts within the interval of 1665 to 228 mg/100 g. It is noteworthy that procyanidin pentamer [EC-(4α-8)-EC-(4α-8)-EC-(4α-8)-EC-(4α-8)-EC] was also found with fairly good amount of 498.8 mg/100 g. Among the galloylated species, most abundant were both, procyanidin dimers B_1_-3-G and B_2_-3′-G with a total amount of 169 mg/100. Apart of catechins and procyanidins, GSE contained appreciable amounts of other phenolic species, such as gallic acid (230 mg/100 g) and ethyl gallate (179 mg/100 g), as well as aminoacids (141 mg/100 g of tryptophan). It is important to note that there were also small peaks that left unidentified, due mostly to insufficiency of their spectral signals and/or incomplete peak resolution. Nevertheless, the amount of 8540 mg/100 g of total procyanidins determined by the acid butanol assay, and 25,098 mg/100 g of total phenols determined by the Folin–Ciocalteu assay suggests that an important part of procyanidins and other phenolic compounds were not assessed by the HPLC method. However, it is known that spectrophotometric methods consistently overestimate the total phenolic concentrations when compared to those determined by HPLC.

### 3.2. Antibiotic Susceptibility

Antibiotic resistance profile and MIC values for *H. pylori* strains are shown in Table 2. Ten strains (71.4%) showed resistance to at least some of the tested antibiotics, while four isolates (28.6%) were susceptible to all tested antibiotics.

Resistance to more than one class of antibiotics was observed in 6 strains (42.9%), being the metronidazole-rifampicin phenotype the prevalent one. Five double resistant strains included three resistant phenotypes (metronidazole-rifampicin, rifampicin-clarithromycin, and clarithromycin-amoxicillin). Only one strain (Hp27) was resistant to four antibiotics (metronidazole-rifampicin-clarithromycin-levofloxacin). No strain was resistant to all of the tested antibiotics. Eight strains were resistant to metronidazole and/or rifampicin, this being the most common phenotype of resistance among all the studied strains (80%). *H. pylori* resistance to clarithromycin, levofloxacin, and amoxicillin was observed in three (21.4%), two (14.3%), and one (7.1%) of the isolates, respectively. No resistance was observed against tetracycline.

### 3.3. Antibacterial Activity

The results of the antibacterial activity of the studied procyanidin rich extract against different clinical strains of *H. pylori* are presented in Table 3. The extract, significantly (*p* ≤ 0.05) inhibited the growth of all isolates tested. GSE exhibited different levels of growth inhibition evidencing a strain-dependent effect. Bacterial growth was totally inhibited in Hp1, Hp2, and Hp3 strains, whereas in the rest of the *H. pylori* strains a reduction of at least 3 log_10_ CFU/mL was observed, except in the strain Hp27, with a reduction of 1.38 log_10_ CFU/mL, demonstrating the strong capacity of the extract to inhibit *H. pylori* growth. Procyanidin-rich extract concentrations as low as 0.125 mg/mL produced a significant inhibition of all isolates (MIC range 0.015–0.125 mg/mL) except in the Hp27 strain, with a MIC of 1 mg/mL. In addition, after % of growth reduction determination, the GSE was bactericidal (or very close to the bactericidal effect) for eight strains, which presented a viability reduction of the initial bacterial inoculum by 99.9%.

## 4. Discussion

*H. pylori* infection caused by antibiotic-resistant strains represents a major public health threat because it is one of the causative agents of gastritis, ulcer and gastric cancer. The eradication rate of *H. pylori* treatment is markedly decreasing in recent years, mainly because of the antibiotic resistance [46]. Currently, six antibiotics are mostly used in combined therapies for *H. pylori* eradication regimes: clarithromycin, amoxicillin, metronidazole, levofloxacin, tetracycline, and rifampicin. Our results showed that the overall prevalence of *H. pylori* antibiotic resistance followed the order: metronidazole = rifampicin > clarithromycin > levofloxacin > amoxicillin > tetracycline. Similar prevalence patterns of *H. pylori* antibiotic resistance have been reported in previous worldwide studies, although these may suffer some modifications depending on variables such as geographical location, age, sex, socioeconomic status, etc. [5,46,47,48].

In the present study, metronidazole and rifampicin showed the highest resistance rates (42.8%). Several studies have reported metronidazole as the most prevalent resistance pattern worldwide [46,49]. In Europe, metronidazole resistance has been estimated in the range of 30–40% [5,6] and this prevalence of resistance in *H. pylori* is even higher in developing countries ranging from 40.5% to 95.4% [50]. Metronidazole has been widely prescribed for infections such as parasitic or female genital infections and could contribute to the high resistance rate found everywhere [51]. Although rifampicin is not commonly used as a first treatment option in *H. pylori* infection, we observed a rising rate of resistance in comparison with previous reports [52,53,54]. However, Regnath et al. [55] reported a considerable increase in resistance to rifampicin from 3.9% to 18.8% between 2002 and 2015 among pediatric patients from southwest Germany. Clarithromycin is recognized as a major antibiotic for *H. pylori* eradication therapy, since it is part of first line triple therapy [56]. However, the occurrence of its resistance is one of the most important forms of antibiotic resistance found among clinically isolated *H. pylori* [57]. The rate of clarithromycin resistance in this study (21%) is consistent with the results reported by others. Although resistance rates are not as high as those of metronidazole in most countries, values around 20% have been reported in several European countries such as Spain, Italy, or Poland [52,56] and resistance rates are increasing worldwide [6,46]. Recently, the World Health Organization (WHO) published a list of bacteria for which new antibiotics are urgently needed [58]. Twelve families were grouped according to their priority (critical, high and medium), and clarithromycin resistant *H. pylori* was included in the high priority group. Fluoroquinolones, such as levofloxacin, are normally used for *H. pylori* eradication in second- or third-line therapies after the failure of clarithromycin treatments. However, levofloxacin resistance has been reported to be 14.1% in a European study [56]. In our study, only two strains were resistant to levofloxacin (14%). On the other hand, it is estimated that among antibiotics used for the treatment of *H. pylori,* resistance rates to amoxicillin and tetracycline are the lowest. The level of resistance to these antibiotics has been reported as very low (< 10%) or even absent in Europe [5,6,51]. In the present work, only one strain was resistant to amoxicillin (*Hp1* strain), and no resistant strains to tetracycline were detected. In previous studies, the resistance rate to at least two antimicrobial agents were reported > 35% for *H. pylori* isolates [59,60]. Our results showed that five of the isolates were resistant to at least two antibiotics (35.7%).

The emergence of *H. pylori* multi-resistant strains to antibiotics has become a serious challenge all over the world. This scenario has drawn the attention of many researchers to the possibility of obtaining antibacterial compounds from other sources [61]. In this regard, grapes and winery by-products (pomace, stems, and seeds) are particularly rich in bioactive phenolic compounds with potential antibacterial properties, and they have proven to be effective against a vast number of microorganisms. For years, it has been considered that the antibacterial activity of a GSE or grape pomace extracts is associated with their high phenolic contents [62]. However, it has been observed that their efficacy against *H. pylori* is not specifically related to the concentration of phenolic compounds, but rather to the type of these compounds and the interaction between them [63]. Indeed, the lack of information on the fine structural composition of these extracts may have influenced their limited effectiveness observed in some animal and human studies [64,65]. However, procyanidin-rich extracts from other plant sources have been shown that they could decrease the incidence of *H. pylori* in humans [66]. Grape seeds contain 60–70% of the extractable phenolic compounds in grapes and the most abundant are flavanols, essentially catechins and procyanidins [67]. Then, the use of grape by-products as a source of catechins and procyanidins could contribute significantly for improving the environmental protection around the winemaking zones [35].

In this study, a procyanidin-rich extract from food-grade winery grape seeds has been obtained using procedures aimed at obtaining a final product with high added value. In this sense, key changes to obtain this extract have been defined in this study that can be summarized as follow: first, selection of intact food-grade grape pomace. This means sweet (not fermented) pomace coming from white wine or grape juice production and treated at hygienic conditions; second, immediate drying of the humid pomace after pressing (from press to dryer); third, substitution of the actual drying systems with indirect heat dryers that can operate at controlled temperatures. In addition, the used extraction procedure with limited heating (40 °C) and prolonged maceration time (1 week) contributes to extract quality. For some commercial GSE, extraction is carried out at accelerated conditions (temperatures between 50 and 80 °C) [43] which favor extraction yields, but also could produce oxidation of procyanidins [68], and afterwards changes in the final composition of the extract. In this way and after sieving, skins and seeds of food-grade quality can be obtained. The procedure described in this study may also help to improve the product homogeneity between batches, which may be a way to prevent large variations in composition affecting the expected antibacterial response.

Concerning the antibacterial activity, the obtained results show that the procyanidin-rich extract obtained under the conditions described here was able to inhibit the growth of all *H. pylori* strains, confirming also the existence of a strain-dependent effect. The behavior observed is consistent with the heterogeneity of the *H. pylori* strains used in this work, which present different profiles of antibiotic sensitivity which could suggest different responses to the antimicrobial action of the extract. However, in all cases the extract caused an inhibition in the growth of *H. pylori*, in a range of MIC from 0.015 to 0.125 mg/mL, which can be considered very interesting MIC values of practical interest for extracts [69]. This relevant bactericidal activity may be associated with its phenolic composition. The results for chemical characterization (Table 1) showed that the high richness in catechin monomers and procyanidin oligomers, positions the extract along with those with higher values for these compounds found in the literature (1900–7143 mg/100 g) [45,70]. Catechins have been shown to possess antimicrobial activity, but this has been considered moderate with respect to those of procyanidins [71]. In particular, procyanidins are considered to have a relevant role in antimicrobial activity, mainly associated with their structure, facilitating the interaction between hydroxyl groups and bacterial membrane. This may be because they allow formation of more branches able to disrupt the bacterial cell membrane [18]. It is important to highlight that the number of hydroxyl groups of the procyanidins increase proportionally with the increase of the degree of polymerization of the procyanidin chains, allowing us to relate, at least partially, the high antibacterial activity of the extract with the high content of procyanidin oligomers (trimers to pentamers).

## 5. Conclusions

In summary, the obtained results have shown a predominance of antibiotic resistance in *H. pylori* which implies that new therapeutics alternatives should be explored. The procyanidin-rich extract used in this study was able to inhibit the growth of all *H. pylori* strains in a MIC range from 0.015 to 0.125 mg/mL. The optimised HPLC-PAD-MS methodology allowed the identification of 43 grape seed compounds and showed that the most of them corresponded to oligomeric procyanidins and catechins. Probably the biggest contribution of this methodology was the quantification of seven procyanidin tetramers, seven procyanidin pentamers, and six galloylated procyanidin dimers, trimers, and tetramers in one analytical run. In this sense, these analytical data are probably the most extensive found in the literature in relation to GSE with antibacterial activity against *H. pylori*. Future studies are needed to identify the individual contribution of each compound, which may help to establish a relationship between composition-activity, contributing to improve the production process of useful antibacterial extracts against *H. pylori*.

## Figures and Tables

**Table 1:** UV absorption and mass spectroscopic data (negative ionization mode) of the identified compounds in the food-grade GSE (contents are expressed as mg/100 g dm).

t_R_ (min)	Compound	[M-H]^−^ (*m/z*)	Product Ions (*m/z*)	Content (mg/100 g)
7.7	3,4,5-THBA (gallic acid)	169.0	125.2, 79.2, 69.1	230 ± 7
13.4	3,4-DHBA (protocatechuic acid)	315.0	153.0, 109.1	11.9 ± 0.3
18.2	*trans*-caftaric acid	311.1	179.0, 135.1	10.1 ± 0.4
23.4	tryptophan	203.1		141 ± 8
23.8	PC_4_	1153.1	865.0, 738.6, 577.0, 574.9, 451.0, 424.9, 289.1, 245.8, 167.1	2.23 ± 0.34
25.1	PC_2_ (B_1_) [EC-(4α-8)-C]	577.0	451.1, 425.0, 407.0, 289.0, 286.9, 271.0, 245.0, 167.1	602 ± 16
25.1	methylgallate	183.0		impurity
25.8	PC_2_ (B_3_) [C-(4α-8)-C]	577.0	575.0, 559.0, 451.0, 425.0, 407.0, 310.6, 289.0, 244.9, 161.1, 139.1	242 ± 11
27.2	C	288.9	271.4, 245.1, 151.0, 149.1, 137.0, 121.1	1665 ± 28
28.2	PC_4_	1153.1	577.8, 245.0, 289.0, 178.8, 161.1	7.48 ± 3.32
28.9	PC_3_	865.0	738.9, 713.0, 577.0, 451.0, 288.0, 244.8	42.4 ± 12.9
29.4	PC_3_	865.0	738.9, 713.1, 576.6, 288.9	35.7 ± 13.6
31.2	PC_3_	865.2	739.0, 713.1, 695.0, 577.0, 575.0, 425.0, 289.9	220 ± 24
32.9	PC_3_	865.3	557.1, 425.0, 289.9	114 ± 25
33.4	PC_4_	1153.1	864.9, 577.0, 451.2, 424.5, 289.1, 287.4, 136.9	104 ± 14
35.2	PC_2_ (B_4_) [C-(4α-8)-EC]	576.8	451.0, 425.0, 407.0, 311.0, 289.9, 245.2	140 ± 13
36.6	PC_3_	865.2	739.1, 712.8, 587.0, 577.0, 425.0, 289.0	28.2 ± 1.7
37.4	PC_2_ (B_2_) [EC-(4α-8)-EC]	577.1	451.0, 425.0, 407.0, 299.0, 289.0, 287.0, 245.1, 161.1	381 ± 12
39.8	PC_5_	1441.1	1153.1, 983.4, 865.0, 577.2, 289.0	55.9 ± 14.1
40.7	EC	289.0	270.8, 166.9, 163.1, 148.9, 145.1, 137.1, 121.3	366 ± 4
40.7	PC_3_	865.0	577.3, 574.8, 425.7	322 ± 11
41.8	PC_3_-G	1017.2	865.0, 729.0, 577.3, 575.1, 441.0, 425.0, 289.0, 245.1,	30.1 ± 2.9
43.0	ethylgallate	197.0	169.0, 151.1, 125.0	179 ± 9
47.7	PC_5_	1441.8	1153.3, 865.0, 713.0, 576.9, 575.0, 451.0, 289.0, 150.9	98.8 ± 12.6
48.2	PC_3_	865.2	847.0, 738.9, 713.0, 695.0, 576.7, 575.0, 406.9	99.0 ± 7.6
50.3	PC_3_ (C_1_) [EC-(4α-8)-EC-(4α-8)-EC]	865.3	740.0, 728.0, 713.1, 695.1, 577.1, 575.0, 559.0, 425.0, 407.0, 286.8	228 ± 25
52.3	PC_2_-G ([ECG-C] (B_1_-3-G) + [EC-ECG] (B_2_-3′-G)	729.0	602.9, 577.0, 559.1, 451.0, 441.0, 424.9, 407.2, 289.0, 168.9	174 ± 8
53.1	PC_3_-2G	1168.5	1017.3, 881.1, 865.2, 727.0, 577.0, 575.0, 440.9, 425.1, 407.1, 290.9, 289.0	29.3 ± 5.8
53.8	PC_4_ [EC-(4α-8)-EC-(4α-8)-EC-(4α-8)-EC]	1153.1	865.0, 576.9, 558.9, 409.4, 289.4, 287.4	165 ± 15
55.9	PC_4_	1153.3	864.9, 862.5, 577.0, 575.0, 425.1, 407.4, 289.4, 286.6	70.3 ± 5.5
55.9	PC_3_-G	1017.3	729.3, 577.0, 575.0, 425.1, 407.4, 289.4, 286.6	impurity
57.0	PC_4_-G	652.7 *	1304.8, 1017.0, 999.1, 729.2, 602.8	53.4 ± 6.3
57.4	PC_5_ [EC-(4α-8)-EC-(4α-8)-EC-(4α-8)-EC-(4α-8)-EC]	1441.1	1152.9, 577.0, 575.0, 558.8, 406.8, 425.0, 289.0	59.4 ± 12.3
58.3	PC_5_	1441.3	1017.4, 865.3	11.4 ± 1.2
58.3	PC_2_ (B5) [EC-(4α-6)-EC]	577.0	450.9, 244.9, 288.9	11.4 ± 1.2
63.0	ECG	441.0	288.9, 169.1	16.2 ± 4.1
64.5	PC_3_-G	1017.3	865.0, 450.8, 286.9	27.3 ± 2.5
65.2	Quercetin-3-O-glucuronide	477.0	301.0, 300, 288.9, 273, 271, 255, 179, 168.9, 150.8, 121	35.6 ± 2.1
66.8	Quercetin-3-O-glucoside	463.0	301.0, 300.0, 271.0, 242.7	5.50 ± 0.26
66.9	PC_4_	1153	865.0, 577.2, 559, 451.2, 425.0, 407.0, 288.8, 244.9	34.6 ± 8.2
67.7	PC_5_	1440.8	1152.0, 864.5, 577.1, 451.8, 559.1, 289.1, 178.9	18.7 ± 0.48
69.7	PC_5_	1441.3	1153.2, 983.7, 865.2, 863.4, 577.2, 450.9, 244.6	11.0 ± 2.3
71.2	PC_5_	1440.9	1153.1, 1135.1, 864.8, 863.0, 713.3, 577.0, 289.0, 270.9	41.3 ± 18.9
	∑ non-galloylated catechins (C + EC)			2031
	∑ ECG			16.2
	**∑ catechins**			**2047**
	∑ PC_2_			1376
	∑ PC_2-_G			174
	**∑ procyanidin dimers**			**1550**
	∑ PC_3_			1089
	∑ PC_3-y_G			86.7
	**∑ procyanidin trimers**			**1176**
	∑ PC_4_			383
	∑ PC_4-y_G			53.4
	**∑ procyanidin tetramers**			**436**
	∑ PC_5_			296
	**∑ procyanidin pentamers**			**296**
	∑ non-galloylated OPC			3440
	∑ galloylated OPC			314
	**∑ OPC**			**3754**
	**∑ flavanols (catechins and OPC)**			**5801**
	∑ HBA			421
	∑ HCA			10.1
	∑ flavonols			41.1
	**∑ non-flavanol phenols**			**472**
	**∑ total individual phenols**			**6273**
	non-phenolic compounds			141
	Total procyanidins (acid butanol assay)			8540 ± 322
	Total phenols (Folin–Ciocalteu assay)			25,098 ± 463

C: catechin; EC: epicatechin; ECG: epicatechin gallate; PCx: procyanidin oligomer (subscript x—number of elemental units, x = 2–5); PC-yG: galloylated procyanidin oligomer, y: number of galloylated units (y = 1,2); HBA: hydroxybenzoic acids; HCA: hydroxycinnamic acids; OPC: oligomer procyanidins. Non-galloylated PC dimers, trimers, tetramers and pentamers are expressed as equivalents of PC_2_ (B_1_). PC-G dimers, trimers and tetramers are expressed as equivalents of ECG; * Double charged ion [M-2H]^2−^.

**Table 2:** Antibiotic resistance and MIC profile of *H. pylori* strains.

Strains	Antibiotic Resistance (MIC) (mg/L)						Total Resistance
	Amoxicillin	Clarithromycin	Levofloxacin	Metronidazole	Rifampicin	Tetracycline	
Hp1	R (0.64)	R (4)	S (0.032)	S (0.032)	S (0.5)	S (<0.016)	2/6
Hp2	S (0.038)	S (0.50)	S (0.125)	R (48)	R (2)	S (0.023)	2/6
Hp3	S (0.032)	R (1.5)	S (0.064)	S (0.75)	R (8)	S (0.023)	2/6
Hp4	S (<0.016)	S (<0.016)	S (<0.002)	R (>256)	R (4)	S (0.38)	2/6
Hp5	S (0.032)	S (0.016)	R (>32)	S (<0.016)	S (0.25)	S (0.094)	1/6
Hp6	S (0.047)	S (<0.016)	S (0.094)	R (96)	S (0.25)	S (0.125)	1/6
Hp7	S (0.047)	S (0.016)	S (0.032)	S (0.094)	S (0.19)	S (0.5)	0/6
Hp8	S (0.047)	S (0.016)	S (0.032)	S (0.094)	S (0.19)	S (0.5)	0/6
Hp9	S (0.047)	S (<0.016)	S (0.094)	R (96)	S (0.25)	S (0.125)	1/6
Hp11	S (0.016)	S (0.125)	S (0.064)	S (0.25)	S (0.75)	S (0.094)	0/6
Hp13	S (<0.016)	S (<0.016)	S (0.064)	S (<0.016)	S (0.032)	S (<0.016)	0/6
Hp14	S (0.023)	S (<0.016)	S (0.19)	S (0.094)	R (3)	S (0.125)	1/6
Hp16	S (<0.016)	S (<0.016)	S (0.094)	R (>256)	R (3)	S (<0.016)	2/6
Hp27	S (<0.016)	R (8)	R (>32)	R (>256)	R (6)	S (0.75)	4/6
Resistant	1/14	3/14	2/14	6/14	6/14	0/14	

**Table 3:** Effects of procyanidin-rich extract on the viable counts of different *H. pylori* strains.

Strains	Control Growth	GSE (2 mg/mL)	Nº log_10_ Reduction (vs Control)	CFU/mL t = 0 h	CFU/mL GSE (2 mg/mL)	% Growth Reduction	MIC (mg/mL)	MIC (log_10_ CFU/mL)
Hp1	8.44 ± 0.11	0.00 ± 0.05 *	8.44	5.60 × 10^6^	0.00	100.0	< 0.015	7.83 ± 0.78
Hp2	7.84 ± 0.23	0.00 ± 0.05 *	7.84	1.39 × 10^6^	0.00	100.0	0.031	6.82 ± 0.01
Hp3	7.05 ± 0.07	0.00 ± 0.05 *	7.05	2.20 × 10^5^	0.00	100.0	0.062	6.41 ± 0.16
Hp4	7.00 ± 0.06	2.83 ± 0.27 *	4.17	2.00 × 10^5^	6.75 × 10^2^	99.7	0.125	4.88 ± 0.21
Hp5	9.03 ± 0.05	3.76 ± 0.11 *	5.27	1.99 × 10^7^	5.70 × 10^3^	100.0	0.031	6.59 ± 0.33
Hp6	7.89 ± 0.09	4.73 ± 0.10 *	3.16	1.60 × 10^6^	5.40 × 10^4^	96.6	0.031	6.98 ± 0.01
Hp7	9.00 ± 0.05	2.30 ± 0.16 *	6.70	1.99 × 10^7^	2.00 × 10^2^	100.0	0.015	8.18 ± 0.07
Hp8	9.11 ± 0.05	1.70 ± 1.41 *	7.41	2.60 × 10^7^	5.00 × 10	100.0	0.062	6.91 ± 0.45
Hp9	8.00 ± 0.05	4.53 ± 0.05 *	3.47	1.99 × 10^6^	3.40 × 10^4^	98.3	0.015	6.51 ± 0.05
Hp11	8.00 ± 0.09	4.76 ± 0.05 *	3.24	1.99 × 10^6^	5.78 × 10^4^	97.1	0.062	7.53 ± 0.01
Hp13	8.32 ± 0.03	2.30 ± 0.16 *	6.02	4.20 × 10^6^	2.00 × 10^2^	100.0	0.062	7.37 ± 0.01
Hp14	7.74 ± 0.05	3.85 ± 0.09 *	3.89	1.12 × 10^6^	7.00 × 10^3^	99.4	0.125	6.15 ± 0.51
Hp16	8.54 ± 0.18	3.67 ± 0.05 *	4.87	6.70 × 10^6^	4.63 × 10^3^	99.9	0.031	6.72 ± 0.03
Hp27	5.95 ± 0.64	4.57 ± 0.05 *	1.38	1.80 × 10^5^	3.75 × 10^4^	79.2	1.0	5.44 ± 0.06

MIC: minimal inhibitory concentration; Values marked with asterisk indicate significant differences compared to the control growth by *t*-test (*p* ≤ 0.05).

## References

[1] Díaz P., Valenzuela Valderrama M., Bravo J., Quest A.F.G. (2018). *Helicobacter pylori* and gastric cancer: Adaptive cellular mechanisms involved in disease progression. Front. Microbiol..

[2] Guevara B., Cogdill A.G. (2020). *Helicobacter pylori*: A review of current diagnostic and management strategies. Dig. Dis. Sci..

[3] Cianci R., Montalto M., Pandolfi F., Gasbarrini G.B., Cammarota G. (2006). Third-Line rescue therapy for *Helicobacter pylori* infection. World J. Gastroenterol..

[4] Lim H.C., Lee Y.J., An B., Lee S.W., Lee Y.C., Moon B.S. (2014). Rifabutin-Based high-dose proton-pump inhibitor and amoxicillin triple regimen as the rescue treatment for *Helicobacter pylori*. Helicobacter.

[5] Savoldi A., Carrara E., Graham D.Y., Conti M., Tacconelli E. (2018). Prevalence of antibiotic resistance in *Helicobacter pylori*: A systematic review and meta-analysis in World Health Organization regions. Gastroenterology.

[6] Thung I., Aramin H., Vavinskaya V., Gupta S., Park J.Y., Crowe S.E., Valasek M.A. (2016). Review article: The global emergence of *Helicobacter pylori* antibiotic resistance. Aliment. Pharmacol. Ther..

[7] MAPA, Spanish Ministry of Agriculture, Fisheries and Nutrition. https://www.mapa.gob.es/es/agricultura/temas/producciones-agricolas/vitivinicultura/default.aspx.

[8] Prodanov M., García Izquierdo C., Alonso Díaz Marta G.L., Lucendo C., Luque Rodríguez S. (2005). Impacto ambiental de la industria vinícola. Parte III. Destilerías de alcohol vínico. Tecnol. Vino.

[9] Oliveira M., Duarte E. (2016). Integrated approach to winery waste: Waste generation and data consolidation. Front. Environ. Sci. Eng..

[10] Prodanov M., Andrés Abellán M., García Morote F.A. (2006). Impacto ambiental de la industria vinícola. La Evaluación del Impacto Ambiental de Proyectos y Actividades Agroforestales.

[11] Ruggieri L., Cadena E., Martínez-Blanco J., Gasol C.M., Rieradevall J., Gabarrell X., Gea T., Sort X., Sánchez A. (2009). Recovery of organic wastes in the Spanish wine industry. Technical, economic and environmental analyses of the composting process. J. Clean. Prod..

[12] Charradi K., Mahmoudi M., Bedhiafi T., Jebari K., El May M.V., Limam F., Aouani E. (2018). Safety evaluation, anti-oxidative and anti-inflammatory effects of subchronically dietary supplemented high dosing grape seed powder (GSP) to healthy rat. Biomed. Pharmacother..

[13] Bagchi D., Garg A., Krohn R.L., Bagchi M., Tran M.X., Stohs S.J. (1997). Oxygen free radical scavenging abilities of vitamins C and E, and a grape seed proanthocyanidin extract In Vitro. Res. Commun. Mol. Pathol. Pharmacol..

[14] Fathima A., Rao J.R. (2016). Selective toxicity of catechin—A natural flavonoid towards bacteria. Appl. Microbiol. Biotechnol..

[15] Ma Y., Ding S., Fei Y., Liu G., Jang H., Fang J. (2019). Antimicrobial activity of anthocyanins and catechins against foodborne pathogens *Escherichia coli* and Salmonella. Food Control.

[16] Silvan J.M., Mingo E., Hidalgo M., de Pascual-Teresa S., Carrascosa A.V., Martinez-Rodriguez A.J. (2013). Antibacterial activity of a grape seed extract and its fractions against *Campylobacter* spp.. Food Control.

[17] Sheng L., Olsen S.A., Hu J., Yue W., Means W.J., Zhu M.J. (2016). Inhibitory effects of grape seed extract on growth, quorum sensing, and virulence factors of CDC “top-six” non-O157 Shiga toxin producing *E. coli*. Int. J. Food Microbiol..

[18] Levy J., Boyer R.R., Neilson A.P., O’Keefe S.F., Chu H.S.S., Williams R.C., Dorenkott M.R., Goodrich K.M. (2017). Evaluation of peanut skin and grape seed extracts to inhibit growth of foodborne pathogens. Food Sci. Nutr..

[19] Sivarooban T., Hettiarachchy N.S., Johnson M.G. (2007). Inhibition of *Listeria monocytogenes* using nisin with grape seed extract on turkey frankfurters stored at 4 and 10 °C. J. Food Prot..

[20] Poveda J.M., Loarce L., Alarcón M., Díaz-Maroto M.C., Alañón M.E. (2018). Revalorization of winery by-products as source of natural preservatives obtained by means of green extraction techniques. Ind. Crop. Prod..

[21] Cosansu S., Juneja V.K., Osoria M., Mukhopadhyay S. (2019). Effect of grape seed extract on heat resistance of *Clostridium perfringens* vegetative cells in sous vide processed ground beef. Food Res. Int..

[22] Santos-Buelga C., García-Viguera C., Tomás-Barberán F.A., Santos-Buelga C., Williamson G. (2003). On-line identification of flavonoids by HPLC coupled to diode array detection. Methods in Polyphenol Analysis.

[23] Saura-Calixto F., Goñi I., Mañas E., Abia R. (1991). Klason lignin, condensed tannins and resistant protein as dietary fibre constituents: Determination in grape pomaces. Food Chem..

[24] Dixon R.A., Xie D.Y., Sharma S.B. (2005). Proanthocyanidin—A final frontier in flavonoid research?. New Phytol..

[25] Sánchez-Patán F., Barroso E., van de Wiele T., Jiménez-Girón A., Martín-Alvarez P.J., Moreno-Arribas M.V., Martínez-Cuesta M.C., Peláez C., Requena T., Bartolomé B. (2015). Comparative In Vitro fermentations of cranberry and grape seed polyphenols with colonic microbiota. Food Chem..

[26] Hümmer W., Schreier P. (2008). Analysis of proanthocyanidins. Mol. Nutr. Food Res..

[27] Lin L.Z., Sun J., Chen P., Monagas M.J., Harnly J.M. (2014). UHPLC-PDA-ESI/HRMSn profiling method to identify and quantify oligomeric proanthocyanidins in plant products. J. Agric. Food Chem..

[28] Li M.N., Wang H.Y., Wang R., Li C.R., Shen B.Q., Gao W., Ping L., Yang H. (2020). A modified data filtering strategy for targeted characterization of polymers in complex matrixes using drift tube ion mobility-mass spectrometry: Application to analysis of procyanidins in the extracts of grape seeds. Food Chem..

[29] Weber H.A., Hodges A.E., Guthrie J.R., O’Brien B.M., Robaugh D., Clarck A.P., Harris R.K., Algaier J.W., Smith C.S. (2007). Comparison of proanthocyanidins in commercial antioxidants: Grape seed and pine bark extracts. J. Agric. Food Chem..

[30] Bernaert H., Allegaert L. (2014). Cocoa Extracts for Use in Providing Skin Benefits. U.S. Patent.

[31] Virot M., Tomao V., Le Bourvellec C., Renard C.M.C.G., Chemat F. (2010). Towards the industrial production of antioxidants from food processing by-products with ultrasound-assisted extraction. Ultrason. Sonochem..

[32] Mateos-Martín M.L., Pérez-Jiménez J., Fuguet E., Torres J.L. (2012). Non-Extractable proanthocyanidins from grapes are a source of bioavailable (epi)catechin and derived metabolites in rats. Br. J. Nutr..

[33] Vorobiev E., Chemat F., Rizvi S.S.H. (2013). Principles of physically assisted extractions and applications in the food, beverage and nutraceutical industries. Separation, Extraction and Concentration Processes in the Food, Beverage and Nutraceutical Industries.

[34] Zhang Y., Liu C., Li J., Qi Y., Li Y., Li S. (2015). Development of ‘‘ultrasound-assisted dynamic extraction’’ and its combination with CCC and CPC for simultaneous extraction and isolation of phytochemicals. Ultrason. Sonochem..

[35] Kalli E., Lappa I., Bouchagier P., Tarantilis P.A., Skotti E. (2018). Novel application and industrial exploitation of winery by-products. Biores. Bioprocess..

[36] Esatbeyoglu T., Wray V., Winterhalter P. (2010). Dimeric procyanidins: Screening for B1 to B8 and semisynthetic preparation of b3, b4, b6, and b8 from a polymeric procyanidin fraction of white willow bark (*Salix alba*). J. Agric. Food Chem..

[37] Prodanov M., Vacas V., Hernández T., Estrella I., Amador B., Winterhalter P. (2013). Chemical characterisation of Malvar grape seeds (*Vitis vinifera* L.) by ultrafiltration and RP-HPLC-PAD-MS. J. Food Compos. Anal..

[38] Grases F., Prieto R.M., Fernandez-Cabot R.A., Costa-Bauza A., Sanchez A.M., Prodanov M. (2015). Effect of consuming a grape seed supplement with abundant phenolic compounds on the oxidative status of healthy human volunteers. Nutr. J..

[39] Li H.J., Deinzer M.L. (2007). Tandem mass spectrometry for sequencing proanthocyanidins. Anal. Chem..

[40] Montero L., Herrero M., Prodanov M., Ibáñez E., Cifuentes A. (2013). Characterization of grape seed procyanidins by comprehensive two-dimensional hydrophilic interaction × reversed phase liquid chromatography coupled to diode array detection and tandem mass spectrometry. Anal. Bioanal. Chem..

[41] Rue E.A., Rush M.D., van Breemen R.B. (2018). Procyanidins: A comprehensive review encompassing structure elucidation via mass spectrometry. Phytochem. Rev..

[42] Porter L.J., Hrstich L.N., Chan B.G. (1985). The conversion of procyanidins and prodelphinidins to cyanidin and delphinidin. Phytochem..

[43] Davidov-Pardo G., Arozarena I., Navarro N., Marin-Arroyo M.R., Segis L. (2015). Microencapsulation of grape seed extracts. Microencapsulation and Microspheres for Food Applications.

[44] Mané C., Souquet J.M., Ollé D., Verriés C., Váran F., Mazerolles G., Cheynier V., Fulcrand H. (2007). Optimization of simultaneous flavanol, phenolic acid, and anthocyanin extraction from grapes using an experimental design: Application to the characterization of champagne grape varieties. J. Agric. Food Chem..

[45] Sá M., Justino V., Spranger M.I., Zhao Y.Q., Hanc L., Sun B.S. (2014). Extraction yields and anti-oxidant activity of proanthocyanidins from different parts of grape pomace: Effect of mechanical treatments. Phytochem. Anal..

[46] De Francesco V., Giorgio F., Hassan C., Manes G., Vannella L., Panella C., Ierardi E., Zullo A. (2010). Worldwide *H. pylori* antibiotic resistance: A systematic review. J. Gastrointest. Liver Dis..

[47] Parsons H.K., Carter M.J., Sanders D.S., Winstanley T., Lobo A.J. (2001). *Helicobacter pylori* antimicrobial resistance in the United Kingdom: The effect of age, sex and socio-economic status. Aliment. Pharmacol. Ther..

[48] Bastos J., Peleteiro B., Barros R., Alves L., Severo M., de Fátima Pina M., Pinto H., Carvalho S., Marinho A., Guimarães J.T. (2013). Sociodemographic determinants of prevalence and incidence of *Helicobacter pylori* infection in Portuguese adults. Helicobacter.

[49] Leitsch D. (2019). A review on metronidazole: An old warhorse in antimicrobial chemotherapy. Parasitology.

[50] Farzi N., Yadegar A., Sadeghi A., Aghdaei H.A., Smith S.M., Raymond J., Suzuki H., Zali M.R. (2019). High prevalence of antibiotic resistance in Iranian *Helicobacter pylori* isolates: Importance of functional and mutational analysis of resistance genes and virulence genotyping. J. Clin. Med..

[51] Alarcon T., Urruzuno P., Martinez M.J., Domingo D., Llorca L., Correa A., Lopez-Brea M. (2017). Antimicrobial susceptibility of 6 antimicrobial agents in *Helicobacter pylori* clinical isolates by using EUCAST breakpoints compared with previously used breakpoints. Enferm. Infecc. Microbiol. Clin..

[52] Alba C., Blanco A., Alarcón T. (2017). Antibiotic resistance in *Helicobacter pylori*. Curr. Opin. Infect. Dis..

[53] Boyanova L., Davidkov L., Gergova G., Kandilarov N., Evstatiev I., Panteleeva E., Mitova I. (2014). *Helicobacter pylori* susceptibility to fosfomycin, rifampin, and 5 usual antibiotics for *H. pylori* eradication. Diagn. Microbiol. Infect. Dis..

[54] Chisholm S.A., Owen R.J. (2009). Frequency and molecular characteristics of ciprofloxacin- and rifampicin-resistant *Helicobacter pylori* from gastric infections in the UK. J. Med. Microbiol..

[55] Regnath T., Raecke O., Enninger A., Ignatius R. (2017). Increasing metronidazole and rifampicin resistance of *Helicobacter pylori* isolates obtained from children and adolescents between 2002 and 2015 in southwest Germany. Helicobacter.

[56] Megraud F. (2013). Current recommendations for *Helicobacter pylori* therapies in a world of evolving resistance. Gut Microbes.

[57] Yeo Y.H., Shiu S.-I., Ho H.J., Zou B., Lin J.-T., Wu M.-S., Liou J.-M., Wu C.-Y. (2018). First-line *Helicobacter pylori* eradication therapies in countries with high and low clarithromycin resistance: A systematic review and network meta-analysis. Gut.

[58] WHO Global Priority List of Antibiotic-Resistant Bacteria to Guide Research, Discovery, and Development of New Antibiotics. https://www.who.int/medicines/publications/global-priority-list-antibiotic-resistant-bacteria/en/.

[59] Hamidi S., Badmasti F., Sadeghpour Heravi F., Safapoor M.H., Tabrizi A.M.A., Ghorbani M., Azizi O. (2020). Antibiotic resistance and clonal relatedness of *Helicobacter pylori* strains isolated from stomach biopsy specimens in northeast of Iran. Helicobacter.

[60] Siavoshi F., Saniee P., Malekzadeh R. (2018). Effective antimicrobial activity of rifabutin against multidrug-resistant *Helicobacter pylori*. Helicobacter.

[61] Parreira P., Duarte M.F., Reis C.A., Martins C.L. (2016). *Helicobacter pylori* infection: A brief overview on alternative natural treatments to conventional therapy. Crit. Rev. Microbiol..

[62] Hassan Y.I., Kosir V., Yin X., Ross K., Diarra M.S. (2019). Grape pomace as a promising antimicrobial alternative in feed: A critical review. J. Agric. Food Chem..

[63] Brown J.C., Huang G.H., Haley-Zitlin V., Jiang X.P. (2009). Antibacterial effects of grape extracts on *Helicobacter pylori*. Appl. Environ. Microbiol..

[64] Brown J.C., Wang J., Kasman L., Jiang X., Haley-Zitlin V. (2010). Activities of muscadine grape skin and quercetin against *Helicobacter pylori* infection in mice. J. Appl. Microbiol..

[65] Chua C.S., Yang K.C., Chen J.H., Liu Y.H., Hsu Y.H., Lee H.C., Huang S.Y. (2016). The efficacy of blueberry and grape seed extract combination on triple therapy for *Helicobacter pylori* eradication: A randomised controlled trial. Int. J. Food Sci. Nutr..

[66] Cires M.J., Wong X., Carrasco-Pozo C., Gotteland M. (2017). The gastrointestinal tract as a key target organ for the health-promoting effects of dietary proanthocyanidins. Front. Nutr..

[67] Badet C., Preedy V.R., Watson R.R., Patel V.B. (2011). Antibacterial activity of grape (*Vitis vinifera*, *Vitis rotundifolia*) seeds. Nuts and Seeds in Health and Disease Prevention.

[68] Escribano-Bailón M.T., Santos-Buelga C., Santos-Buelga C., Williamson G. (2003). Polyphenol extraction from foods. Methods in Polyphenol Analysis.

[69] Ríos J.L., Recio M.C. (2005). Medicinal plants and antimicrobial activity. J. Ethnopharmacol..

[70] Sica V.P., Mahony C., Baker T.R. (2018). Multi-Detector characterization of grape seed extract to enable in silico safety assessment. Front. Chem..

[71] Mayer R., Stecher G., Wuerzner R., Colonia Silva R., Sultana T., Trojer L., Feuerstein I., Krieg C., Abel G., Popp M. (2008). Proanthocyanidins: Target compounds as antibacterial agents. J. Agric. Food Chem..

